# Risk factors for thromboembolic events in patients with paroxysmal nocturnal hemoglobinuria (PNH): a nested case–control study in the International PNH Registry

**DOI:** 10.1007/s00277-023-05402-3

**Published:** 2023-09-05

**Authors:** Britta Höchsmann, Regis Peffault de Latour, Anita Hill, Alexander Röth, Timothy Devos, Christopher J. Patriquin, Wen-Chien Chou, Deepak Jain, Ke Zu, Chuntao Wu, Jong Wook Lee

**Affiliations:** 1 Institute of Transfusion Medicine, University of Ulm, and Institute for Clinical Transfusion Medicine and Immunogenetics, German Red Cross Blood Transfusion Service Baden-Württemberg-Hessen and University Hospital Ulm, Helmholtzstraße 10, 89081 Ulm, Germany; 2 Bone Marrow Transplantation Unit, Saint Louis Hospital, Assistance Publique-Hôpitaux de Paris, Paris, France; 3 French Reference Center for Aplastic Anemia and Paroxysmal Nocturnal Hemoglobinuria, Saint Louis Hospital and University Paris Diderot, Paris, France; 4 Severe Aplastic Anemia Working Party of the European Group for Blood and Marrow Transplantation, Leiden, The Netherlands; 5 Department of Hematology, Leeds Teaching Hospitals, Leeds, England UK; 6 Alexion, AstraZeneca Rare Disease, Leeds, UK; 7 Department of Hematology and Stem Cell Transplantation, West German Cancer Center, University Hospital Essen, Essen, Germany; 8 Department of Hematology, University Hospitals Leuven and Department of Microbiology and Immunology, Laboratory of Molecular Immunology (Rega Institute), KU Leuven, Leuven, Belgium; 9 Division of Medical Oncology & Hematology, University of Toronto, Toronto, ON Canada; 10 Department of Laboratory Medicine, National Taiwan University Hospital, Taipei, Taiwan; 11 Alexion, AstraZeneca Rare Disease, Boston, MA USA; 12 Department of Hematology, Seoul St. Mary’s Hospital, The Catholic University of Korea, Seoul, Republic of Korea

**Keywords:** Paroxysmal nocturnal hemoglobinuria, Cohort study, Thromboembolism, Multivariable analysis, Risk factors

## Abstract

The objective of this analysis was to identify risk factors for thromboembolic events (TE) in patients with paroxysmal nocturnal hemoglobinuria (PNH) who were not treated with C5 inhibitors. Patients with PNH and a history of ≥ 1 TE at enrollment in the International PNH Registry (NCT01374360; registration date, January 2011) were each matched with up to 5 patients without TE. Multivariable analysis was performed with the following variables: percentage glycosylphosphatidylinositol (GPI)–negative cells, high disease activity (HDA), non-TE major adverse vascular event history, and recent anticoagulation. Of 2541 eligible patients, 57 with TE and 189 matched controls were analyzed. Multivariable analysis (odds ratio [95% CI]) identified the following factors as being associated with increased thrombotic risk: patients with no history of TE (with recent anticoagulation, 9.30 [1.20–72.27]), patients with history of TE (with recent anticoagulation, 8.91 [0.86–92.62]; without recent anticoagulation, 5.33 [0.26–109.57]), patients with ≥ 30% GPI-negative granulocytes (≥ 30% to < 50%, 4.94 [0.54–45.32]; ≥ 50%, 1.97 [0.45–8.55]), or patients with lactate dehydrogenase (LDH) ratio ≥ 1.5 × upper limit of normal (ULN) plus ≥ 2 HDA criteria (2–3 criteria, 3.18 [0.44–23.20]; ≥ 4 criteria, 3.60 [0.38–33.95]). History of TE, ≥ 30% GPI-negative granulocytes, and LDH ratio ≥ 1.5 × ULN with ≥ 2 HDA criteria are TE risk factors for patients with PNH. These findings will aid physicians by providing important clinical and laboratory risk factors that can be used to identify and manage patients with PNH who are at risk of developing TE.

**Supplementary Information:**

The online version contains supplementary material available at 10.1007/s00277-023-05402-3.

## Introduction

Paroxysmal nocturnal hemoglobinuria (PNH) is a rare, acquired, clonal hematopoietic stem cell disorder caused by a somatic mutation of the phosphatidylinositol glycan class A (*PIG-A*) gene [1–4]. This defect in *PIG-A* reduces surface expression of the glycosylphosphatidylinositol (GPI)–anchored complement regulatory proteins CD55 and CD59 on hematopoietic cells (ie, erythrocytes, platelets, and leukocytes) and leads to terminal complement–mediated intravascular hemolysis and increased risk of thrombosis [5, 6]. Patients with PNH present with diverse clinical manifestations, the most common being chronic intravascular hemolysis, fatigue, and dyspnea [7–9]. Thromboembolic events (TEs) are the leading cause of death in patients with PNH, accounting for 40% to 67% of deaths before the complement inhibition era [10–12], with a 5‐year mortality rate of approximately 30% [13]. The risk of incident thrombosis increases over time if targeted therapy is not initiated [5, 12]. The numerous mechanisms of TE in patients with PNH are complex and include complement-mediated platelet and leukocyte activation, intravascular hemolysis, nitric oxide depletion, impairment of the fibrinolytic system, and increased inflammatory mediators [14, 15]. It is important to identify and characterize risk factors predictive of TE because they can assist in improved monitoring and early intervention, which may lead to better outcomes for patients [16].

Some risk factors for TE in patients with PNH have been described in the literature. Data from 2 studies showed that the proportion of GPI-negative granulocytes (> 50% and ≥ 61% in the first and second study, respectively) is significantly associated with a risk of TE [10, 17]. An analysis of data from the Korean PNH Registry found significant correlations between TE risk and elevated lactate dehydrogenase (LDH) and between TE risk and clinical symptoms such as abdominal pain, chest pain, dyspnea, or hemoglobinuria using multivariable analyses [18]. Importantly, anemia and transfusion dependence were not identified as risk factors for TE. The Korean PNH Registry data also showed a correlation between risk of TE and percentage of GPI-negative granulocytes but with no evidence of association between specific GPI-negative granulocyte thresholds and highlighted that even patients with a smaller population of PNH granulocytes (< 20%) were at risk for thrombosis. Other studies have found that patients with a higher proportion of PNH granulocytes and elevated LDH are more likely to have a history of TE [7, 8]. Furthermore, history of TE at diagnosis has been identified as a risk factor for subsequent TE [19]. While previous investigations have advanced the understanding between TE and associated risk factors, this more comprehensive analysis using international real-world data from a more diverse dataset can add further insights in identifying relevant factors affecting risk for TE in patients with PNH.

The International PNH Registry (NCT01374360) is an ongoing, observational cohort study containing the largest database of safety, quality-of-life, and outcomes data from treated and untreated patients with PNH [20]. In the current study, we analyzed patient data from the Registry to identify risk factors for TE and evaluate the odds of TE in the presence of specific disease characteristics and clinical parameters.

## Methods

### Patient population

Patient data from the International PNH Registry with a cutoff date of July 8, 2019, were eligible for analysis. This analysis included data from patients who were not treated with complement inhibitors at enrollment and had an incident TE after enrollment, a non-zero follow-up time, and documented information regarding their birthdate, sex, enrollment date, treatment status, and country. Untreated patients who had ≥ 1 TE after enrollment in the Registry were defined as TE cases. The first TE after enrollment in the Registry was defined as the index event for the case, and the date at which it occurred was defined as the index date.

A matched case–control design nested in the Registry was employed to reduce confounding. For each TE case, up to 5 untreated controls who did not experience a TE event at the time the index case occurred were randomly selected, matching on age (± 5 years at index date), sex, country, and history of bone marrow failure (BMF). TE cases that could not be matched with any controls were excluded from the analysis. For covariates included in the analysis, absent values were marked as missing.

### Assessments

Patient demographics and clinical parameters before and immediately preceding the TE index date were gathered for both TE cases and controls. All values for the assessed risk factors were based on the most recently reported data point in the 6 months before the TE index date except for history of BMF (measured from PNH diagnosis to the index date) and physician-documented symptoms (assessed 1–6 months before the TE index date). History of TEs and other major adverse vascular events (MAVEs) was classified based on records between the date of PNH diagnosis and the index date. MAVEs included TEs (thrombophlebitis/deep vein thrombosis, renal vein thrombosis, renal arterial thrombosis, mesenteric/visceral vein thrombosis, mesenteric/visceral arterial thrombosis, hepatic/portal vein thrombosis, dermal thrombosis, acute peripheral vascular disease occlusion, cerebral arterial occlusion/cerebrovascular accident, cerebral venous occlusion, or pulmonary embolus) and non-TEs (nontraumatic amputation, nondiabetic amputation, myocardial infarction, transient ischemic attack, unstable angina, nontraumatic gangrene, nondiabetic gangrene, or “other”).

Univariable analyses were conducted for TE risk associated with the following risk factors: GPI-negative granulocytes, GPI-negative erythrocytes, LDH ratio, high disease activity (HDA; defined as LDH ratio ≥ 1.5 × upper limit of normal [ULN] and ≥ 1 of the following 1 to 6 months before the TE index date: anemia [hemoglobin < 10 g/dL], abdominal pain, dyspnea, dysphagia, fatigue, hemoglobinuria, or male erectile dysfunction), LDH ratio ≥ 1.5 × ULN and ≥ 1 HDA symptom, PNH-related symptoms (ie, abdominal pain, backache, hemoglobinuria, dysphagia, fatigue, headache, male erectile dysfunction, and dyspnea), recent anticoagulation (ie, within 6 months), history of TEs, and history of MAVEs. Despite the inclusion of the gender-specific variable of erectile dysfunction, based on the approach to modeling, a gender bias should not have been introduced. Based on existing literature from studies that explored risk factors for TEs in PNH, the following variables were selected for the multivariable analysis [7, 8, 10, 17, 18]: proportion of GPI-negative granulocytes, LDH ratio ≥ 1.5 × ULN and ≥ 1 HDA criterion, history of non-TE MAVEs, and recent anticoagulation.

### Statistical analyses

Descriptive analyses of patient characteristics and clinical parameters included mean and SD for continuous variables and frequencies and percentages for categorical variables. Univariable and multivariable conditional logistic regressions were used in the matched subpopulation to estimate odds ratios (ORs) and associated 95% Wald’s CIs for incident TE.

## Results

### Patient demographics and disease burden

**Fig. 1:**
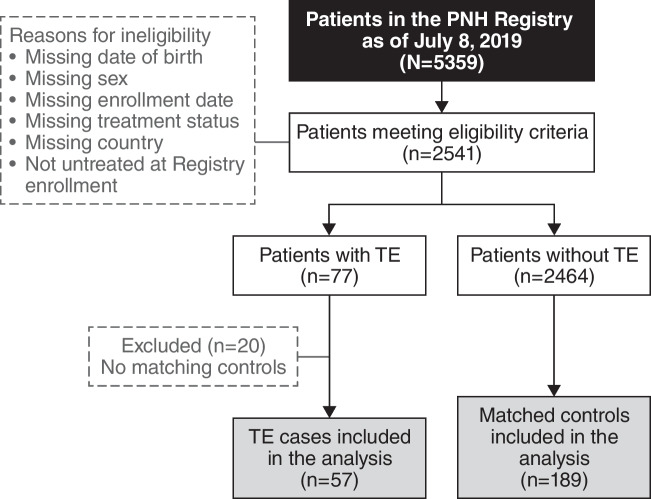
TE case and control selection from the International PNH Registry. PNH, paroxysmal nocturnal hemoglobinuria; TE, thromboembolic event

**Fig. 2:**
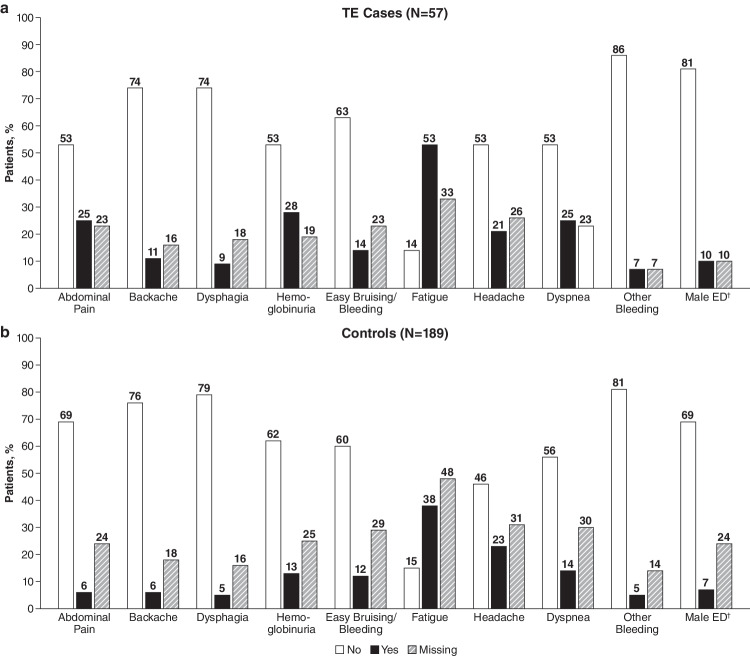
Physician-reported symptoms within 6 months before the index date. (**a**) TE cases. (**b**) Controls. ^†^TE cases (n = 31), controls (n = 103). ED, erectile dysfunction; TE, thromboembolic event

As of the July 8, 2019, cutoff date, 77 of 2541 patients in the Registry eligible for this study had TE. Of these, 57 met the inclusion criteria for TE cases, and 189 controls were matched to these cases (Fig. 1). The remaining 20 cases of TE did not have matching controls and were therefore excluded. Demographics and patient characteristics were similar between TE cases and controls: most patients were European (77.6%) and male (54.5%), and the mean age of both groups at the index date was 47 years (Table 1). Fewer TE cases had a history of BMF compared with controls (68.4% and 78.8%, respectively). Fewer TE cases versus controls had < 10% and ≥ 10% to < 30% GPI-negative granulocytes (< 10%, 12.3% vs 22.8%; ≥ 10% to < 30%, 1.8% vs 8.5%). More TE cases than controls had ≥ 50% GPI-negative granulocytes (29.8% vs 13.2%), LDH ratio ≥ 1.5 × ULN (38.6% vs 21.7%), HDA (33.3% vs 15.3%), and a history of TEs (21.1% vs 5.8%) or MAVEs (24.6% vs 10.6%). Among patients with documented use of anticoagulation, 67.0% of TE cases (n = 18/27) and 76.2% of controls (n = 16/21) had no prior history of TE. Most of these patients initiated anticoagulation before their most recent TE. The most common physician-documented symptoms experienced by the TE cases vs the controls were fatigue (52.6% vs 37.6%), hemoglobinuria (28.1% vs 12.7%), abdominal pain (24.6% vs 6.3%), and dyspnea (24.6% vs 13.8%; Fig. 2).

**Table 1:** Patient characteristics and baseline clinical parameters^†^

Parameters	TE Cases (n = 57)	Controls (n = 189)
Patient Demographics		
Sex, n (%)		
Male	31 (54.4)	103 (54.5)
Female	26 (45.6)	86 (45.5)
Region, n (%)		
Europe	42 (73.7)	149 (78.8)
Asia	7 (12.3)	23 (12.2)
North America	6 (10.5)	15 (7.9)
Australia/New Zealand	2 (3.5)	2 (1.1)
Age at index date, mean (SD)	46.8 (17.1)	47.1 (16.0)
Years from PNH diagnosis to index date, mean (SD)	7.4 (8.4)	7.4 (8.5)
History of BMF, n (%)	39 (68.4)	149 (78.8)
Clinical Parameters		
Percent GPI-negative granulocytes, n (%)		
< 10%	7 (12.3)	43 (22.8)
≥ 10% to < 30%	1 (1.8)	16 (8.5)
≥ 30% to < 50%	3 (5.3)	6 (3.2)
≥ 50%	17 (29.8)	25 (13.2)
Missing	29 (50.9)	99 (52.4)
Percent GPI-negative erythrocytes (type II and III), n (%)		
< 10%	13 (22.8)	55 (29.1)
≥ 10%	16 (28.1)	28 (14.8)
Missing	28 (49.1)	106 (56.1)
LDH ratio (× ULN), n (%)		
< 1.5	15 (26.3)	71 (37.6)
≥ 1.5	22 (38.6)	41 (21.7)
Missing	20 (35.1)	77 (40.7)
HDA^‡^		
No	18 (31.6)	83 (43.9)
Yes	19 (33.3)	29 (15.3)
Missing	20 (35.1)	77 (40.7)
LDH ratio (× ULN), n (%) and HDA criteria		
LDH < 1.5 × ULN and no HDA criteria	4 (7.0)	27 (14.3)
LDH ≥ 1.5 × ULN with 0-1 HDA criterion	5 (8.8)	22 (11.6)
LDH ≥ 1.5 × ULN with 2–3 HDA criteria	10 (17.5)	13 (6.9)
LDH ≥ 1.5 × ULN with ≥ 4 HDA criteria	7 (12.3)	6 (3.2)
Missing	31 (54.4)	121 (64.0)
History of MAVE, n (%)^§^		
No	42 (73.7)	169 (89.4)
Yes	14 (24.6)	20 (10.6)
Missing	1 (1.8)	0 (0.0)
History of non-TE MAVE, n (%)^§^		
No	49 (86.0)	177 (93.7)
Yes	7 (12.3)	12 (6.3)
Missing	1 (1.8)	0 (0.0)
History of TE, n (%)^§^		
No	44 (77.2)	178 (94.2)
Yes	12 (21.1)	11 (5.8)
Missing	1 (1.8)	0 (0.0)
Recent anticoagulation, n (%)^¶^		
No	15 (26.3)	63 (33.3)
Yes	27 (47.4)	21 (11.1)
Missing	15 (26.3)	105 (55.6)

^†^The index date is the first TE event after enrollment in the Registry. Data are based on assessments within 6 months before the TE index date. ^‡^HDA defined as LDH ratio ≥ 1.5 × ULN, hemoglobin < 10 g/dL, and one of the following symptoms 6 months before and including the index date: abdominal pain, dyspnea, dysphagia, fatigue, hemoglobinuria, or erectile dysfunction. ^§^Assessments based on the entire patient history from PNH diagnosis to index date. ^¶^Recent is defined as within 6 months before the index date. BMF, bone marrow failure; GPI, glycosylphosphatidylinositol; HDA, high disease activity; LDH, lactate dehydrogenase; MAVE, major adverse vascular event; PNH, paroxysmal nocturnal hemoglobinuria; TE, thromboembolic event; ULN, upper limit of normal

### Univariable and multivariable analyses

**Fig. 3:**
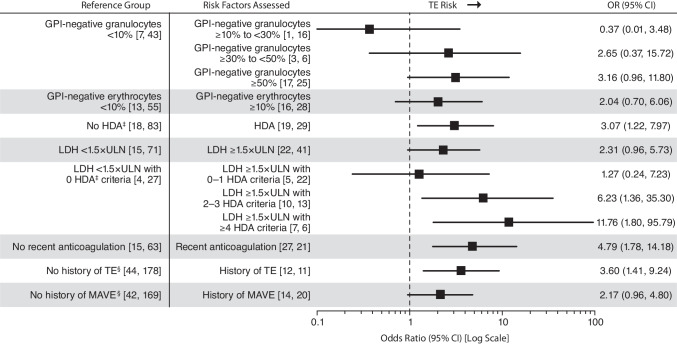
Univariable analysis of TE risk by laboratory and clinical parameters. Data are based on assessments within 6 months before the TE index date. Patient numbers are reported as [TE cases, controls] per parameter. Exact OR and estimated CIs are from univariable conditional logistic regression model of risk of TE event. The increased risk of TE observed in patients receiving anticoagulation with no history of TE likely reflects prescribing decisions based on physician assessment of TE risk (ie, primary prophylaxis) rather than contributing to risk itself and should be interpreted accordingly. ^‡^HDA defined as LDH ratio ≥ 1.5 × ULN, hemoglobin < 10 g/dL, and one of the following symptoms 6 months before and including the index date: abdominal pain, dyspnea, dysphagia, fatigue, hemoglobinuria, or erectile dysfunction. ^§^Assessments based on the entire patient history from PNH diagnosis to index date. GPI, glycosylphosphatidylinositol; HDA, high disease activity; LDH, lactate dehydrogenase; MAVEs, major adverse vascular events; OR, odds ratio; TE, thromboembolic event; ULN, upper limit of normal

**Fig. 4:**
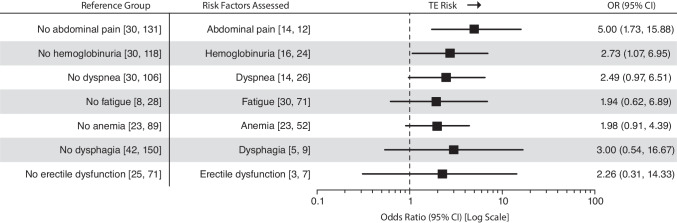
Univariable analysis of TE risk by physician-documented symptom. Data are based on assessments within 6 months before the TE index date. Patient numbers are reported as [TE cases, controls] per parameter. Exact OR and estimated CIs are from univariable conditional logistic regression model of risk of TE event. OR, odds ratio; TE, thromboembolic event

Univariable analyses showed that patients with LDH ≥ 1.5 × ULN and ≥ 4 HDA criteria had the greatest increased risk of TE compared with patients with LDH < 1.5 × ULN and no criteria (OR, 11.76; 95% CI, 1.80–95.79; Fig. 3). Other laboratory and clinical parameters associated with an increased risk of TE over their respective reference groups were LDH ≥ 1.5 × ULN and 2 to 3 HDA criteria (OR, 6.23; 95% CI, 1.36–35.30), recent anticoagulation (OR, 4.79; 95% CI, 1.78–14.18), history of TE (OR, 3.60; 95% CI, 1.41–9.24), HDA (OR, 3.07; 95% CI, 1.22–7.97), and history of MAVEs (OR, 2.17; 95% CI, 0.96–4.80). Compared to the reference groups, increased TE risk was also seen in patients with physician-documented symptoms of abdominal pain (OR, 5.00; 95% CI, 1.73–15.88), dysphagia (OR, 3.00; 95% CI, 0.54–16.67) and hemoglobinuria (OR, 2.73; 95% CI, 1.07–6.95) (Fig. 4).

**Fig. 5:**
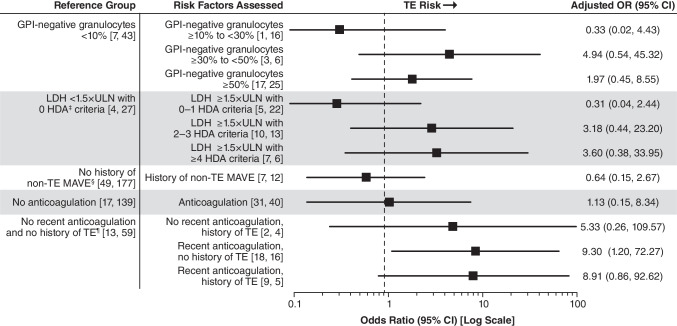
Multivariable analysis of TE risk by laboratory and clinical parameters. Data are based on assessments within 6 months before the TE index date. Patient numbers are reported as [TE cases, controls] per parameter. OR and 95% CIs are Wald’s estimates from multivariable conditional logistic regression model of risk of TE event. The increased risk of TE observed in patients receiving anticoagulation with no history of TE likely reflects prescribing decisions based on physician assessment of TE risk (ie, primary prophylaxis) rather than contributing to risk itself and should be interpreted accordingly. ^‡^HDA defined as LDH ratio ≥ 1.5 × ULN, hemoglobin < 10 g/dL, and one of the following symptoms 6 months before and including the index date: abdominal pain, dyspnea, dysphagia, fatigue, hemoglobinuria, or erectile dysfunction. ^§^Assessments based on the entire patient history from PNH diagnosis to index date. ^¶^Recent is defined as within 6 months before the index date. GPI, glycosylphosphatidylinositol; HDA, high disease activity; LDH, lactate dehydrogenase; MAVE, major adverse vascular event; OR, odds ratio; TE, thromboembolic event; ULN, upper limit of normal

Multivariable analysis identified the following as being associated with increased risk of TE: no history of TE with recent anticoagulation (OR, 9.30 [95% CI, 1.20–72.27]), history of TE with recent anticoagulation (OR, 8.91; 95% CI, 0.86–92.62), history of TE without recent anticoagulation (OR, 5.33; 95% CI, 0.26–109.57), ≥ 30% to < 50% GPI-negative granulocytes (OR, 4.94; 95% CI, 0.54–45.32); ≥ 50% GPI-negative granulocytes (OR, 1.97; 95% CI, 0.45–8.55); LDH ratio ≥ 1.5 × ULN and 2 to 3 HDA criteria (OR, 3.18; 95% CI, 0.44–23.20); LDH ratio ≥ 1.5 × ULN and ≥ 4 HDA criteria (OR, 3.60; 95% CI, 0.38–33.95) (Fig. 5). Unlike the univariable analysis, recent anticoagulation alone was not associated with TE risk (OR, 1.13; 95% CI, 0.15–8.34); history of non-TE MAVE was also not associated with TE risk (OR, 0.64; 95% CI, 0.15–2.67).

## Discussion

This observational PNH Registry analysis used data from an international real-world setting and identified history of TE, ≥ 30% GPI-negative granulocytes, and LDH ≥ 1.5 × ULN plus ≥ 2 HDA criteria as risk factors for TE using multivariable analysis. Notably, increased TE risk was observed in patients with a prior history of TE regardless of anticoagulation use. Moreover, this analysis found that increased TE risk was also associated with a set of physician-documented symptoms, including abdominal pain, hemoglobinuria, and dysphagia, which may be symptomatic markers of hemolysis or nitric oxide sequestration. Together, these results provide important clinical and laboratory risk factors that can be used to identify and manage patients with PNH who are at risk of developing TE.

Current recommendations for the management of thrombosis in PNH advise that all patients unable to receive complement inhibition with > 50% GPI-negative granulocytes or with additional risk factors (eg, pregnancy, hemolytic crisis) should commence anticoagulation for primary thromboprophylaxis assuming there are no contraindications [4, 13, 17, 21]. In some countries, anticoagulation is recommended in all patients with proportion of > 50% GPI-negative granulocytes [17, 21]. For patients without access to complement inhibition therapy, it might be an option to lower the threshold for prophylactic anticoagulation from > 50% GPI-negative granulocytes to ≥ 30% GPI-negative granulocytes. Due to the possible serious complications and the debated efficiency, this must be carefully discussed on the basis of a larger patient group. More research is needed to clearly show the benefits of prophylactic anticoagulation on the risk of TE in patients with ≥ 30% GPI-negative granulocytes, particularly if they have other biomarkers (eg, LDH ≥ 1.5 × ULN) suggesting an increased thrombotic risk [18]. For those with access, complement inhibition therapy might be beneficial for patients with ≥ 30% GPI-negative granulocytes and LDH < 1.5 × ULN. As the small sample size of this group lacks power to detect statistical significance, further data are needed to confirm this assumption. Patients not treated with a terminal complement inhibitor (eg, eculizumab, ravulizumab) remain at an increased risk of thrombosis even with full-dose anticoagulation [22]. Patients receiving primary prophylaxis who commence complement inhibition may be able to stop anticoagulation after intravascular hemolysis is controlled (as evidenced by LDH < 1.5 × ULN), providing they have not experienced a TE in the interim [13]. Based on our analysis and previous studies suggesting that the use of anticoagulants may not be protective for the management of TE in patients with PNH [18, 22], it remains unclear whether anticoagulation alone is sufficiently effective in addressing prothrombic risk. In the current analysis, increased risk of TE was observed in patients receiving anticoagulation with no history of TE; this is expected, likely reflecting prescribing decisions based on physician assessment of increased TE risk and the use of primary prophylaxis, rather than adversely contributing to risk itself and should be interpreted accordingly.

Our data are in alignment with those of a prior multivariable analysis from the South Korean PNH Registry (N = 301) [18]. In that study, untreated patients with PNH who had LDH levels ≥ 1.5 × ULN at diagnosis were at a significantly higher risk for TE than patients with LDH < 1.5 × ULN (OR, 7.0; *P* = 0.013). Risk of TE increased with intravascular hemolysis, abdominal pain, chest pain, dyspnea, and hemoglobinuria, but not anemia. In a previous analysis of the baseline characteristics and disease burden of patients enrolled in the International PNH Registry, a significantly larger proportion of untreated patients with LDH ≥ 1.5 × ULN at enrollment vs LDH < 1.5 × ULN had a history of TE (16% vs 8%, *P* < 0.001) [8]. Our analysis using data from the International PNH Registry also identified intravascular hemolysis (LDH ≥ 1.5 × ULN), abdominal pain, and hemoglobinuria, but not anemia and dyspnea, as potential TE risk factors. Lee et al*.* reported a correlation between risk of TE and percentage of GPI-negative granulocytes but with no evidence of association between specific GPI-negative granulocyte thresholds, concluding that LDH may be more predictive of TE risk [18]. Likewise, in the current analyses, the OR point estimates for > 30% PNH granulocytes were associated with increased risk of TE; however, we found a correlation but no statistical significance between proportion of GPI-negative granulocytes and risk of TE. Thus, our analysis extends the findings of the previous single-country analysis to a more diverse, international population.

In contrast, a long-term analysis of 163 PNH patients not treated with complement inhibitors revealed a significantly increased 10-year risk of thrombosis in those with > 50% (44%) vs < 50% (6%) GPI-negative granulocytes [17]. In patients with > 50% GPI-negative granulocytes, a significant reduction in 10-year thrombotic risk was also seen in those receiving warfarin primary prophylaxis. In 2014, Schrezenmeier et al*.* analyzed patient data from the International PNH Registry and found a notable association between history of TEs and proportion of GPI-negative granulocytes (*P* < 0.001), although all patients were at risk of experiencing a TE independent of their proportion of GPI-negative granulocytes [8]. Additionally, Moyo et al*.* found that for each 10% increase in GPI-negative granulocytes, the OR of thrombosis increased by 1.64 (*P* = 0.008) [10]. These discrepancies between our study and the previous reports could be due to the small sample sizes in the proportion of GPI-negative granulocyte subgroups in our analysis. Further research could better elucidate the nature of the relationship between the proportion of GPI-negative granulocytes or monocytes and risk of TE.

There are limitations of this analysis worth noting. This analysis utilized an observational data set, and thus values of potential risk factors for TE are missing in some patients, at times up to approximately 50%. Although the pool of individuals with PNH used in this analysis is the largest to date, only a subset of patients met the TE case inclusion criteria. Of note, prior treatment with complement inhibitors was exclusionary, and thus patients potentially at highest risk were not included in the analysis, which may have influenced the results. Further, the study did not control for traditional TE risk factors such as smoking and obesity [23]. We were also unable to identify the reasons that patients with a history of TE were not receiving anticoagulation. Despite these limitations, many factors that were significantly associated with increased TE risk were identified from the analysis. The strengths of this study include a robust design for evaluation of one of the most life-threatening outcomes in PNH and its relationship to identifiable (and several modifiable) risk factors, and an increased generalizability with an international patient population.

## Conclusions

This retrospective analysis from the International PNH Registry identified likely risk factors associated with TE, the most serious complication and leading cause of death for patients with PNH, and indicated that patients with PNH are at an increased risk of TE if they have a prior history of TE, ≥ 30% GPI-negative granulocytes, or LDH ≥ 1.5 × ULN plus ≥ 2 HDA criteria. The findings of this analysis were based on data derived from a broad global database and confirm the results of previous studies. Further study to identify and monitor risk factors for TE in patients with PNH is warranted to better inform treatment decisions and improve patient outcomes.

## Supplementary Information

Below is the link to the electronic supplementary material.

Supplementary file1 (DOCX 27.9 KB)

## Data Availability

Alexion, AstraZeneca Rare Disease will consider requests for disclosure of clinical study participant-level data provided that participant privacy is assured through methods such as data de-identification, pseudonymization, or anonymization (as required by applicable law), and if such disclosure was included in the relevant study informed-consent form or similar documentation. Qualified academic investigators may request participant-level clinical data and supporting documents (statistical analysis plan and protocol) pertaining to Alexion-sponsored studies. Further details regarding data availability and instructions for requesting information are available in the Alexion Clinical Trials Disclosure and Transparency Policy at http://alexion.com/our-research/research-and-development. Link to data-request form: https://alexion.com/contact-alexion/medical-information.
