# Supplementary material for: Risk factors for thromboembolic events in patients with paroxysmal nocturnal hemoglobinuria (PNH): a nested case–control study in the International PNH Registry
Source: Ann Hematol. 2023 Sep 5;102(11):2979–88. doi: 10.1007/s00277-023-05402-3 (PMC10567964; doi:10.1007/s00277-023-05402-3)
Supplement: Supplementary file 1 — Supplementary file1 (DOCX 27.9 KB) [file 277_2023_5402_MOESM1_ESM.docx]

| This article reports risk factors that may cause patients with paroxysmal nocturnal hemoglobinuria, also called PNH, to have blood clots that block blood vessels (thromboembolic events, also known as TEs).   - - This study looked at data from people with PNH who are enrolled in the International PNH Registry.   - People with PNH sometimes experience blood clots, which may lead to death.   - It is important to understand what disease characteristics may make people with PNH more likely to have a blood clot so that patients and their physicians can make informed treatment decisions. |
| --- |
| **What is PNH?**   - - PNH is a rare blood disease that can be life threatening.   - The complement system is a part of the body’s natural defense against invading organisms such as bacteria and viruses. It targets them for destruction.     - People with PNH have some blood cells that do not make a protein called GPI. Without GPI, proteins that protect cells from the complement system are missing on the surface of red blood cells, white blood cells, and platelets.   - Without protective proteins, there is uncontrolled complement activity on the surface of these cells, resulting in the breakdown of the cells.   - In patients with PNH, too much complement activity activates their white blood cells and platelets while causing their red blood cells to break apart inside of blood vessels (called intravascular hemolysis).   - These broken red blood cells plus the activated white blood cells and platelets can result in blood clots that may block blood vessels.   - These blood clots are the most common cause of death in people with PNH. |
| **What did this study investigate?**   - - This study used real-world data from the International PNH Registry. It is the largest collection of anonymized clinical data on people with PNH that is gathered over time.   - Data from the International PNH Registry were used to look for risk factors that may make patients with PNH more or less likely to have blood clots.   - The researchers looked at the following characteristics to see if they were risk factors for PNH:     - History of blood clots and other harmful vascular issues including heart attack or stroke     - Proportion of blood cells that do not have the GPI protein on the surface (also called PNH clone size)     - Levels of an enzyme called lactate dehydrogenase (also known as LDH)     - PNH-related symptoms (abdominal or belly pain, backache, high hemoglobin levels in the urine [also called hemoglobinuria], difficulty swallowing, fatigue, headache, erectile dysfunction, difficulty breathing)     - Recent use of anticoagulation medicine. These drugs are sometimes called blood thinners. They help prevent blood clots from forming or getting bigger. |
| **What are the main findings and conclusions from the study?**   - - This study found that patients with the following characteristics were at greater risk of having a blood clot in the future:     - Having a history of blood clots     - Having more than 30% of blood cells without GPI, in other words, a clone size of 30% or more     - Having higher than normal levels of LDH plus 2 or more PNH-related symptoms   - Patients with these characteristics should be very carefully monitored for blood clots. Identifying these characteristics might help patients and their physicians consider current treatment options. |
| **This summary is based on the following research article**   - - Höchsmann B, et al. Risk Factors for Thromboembolic Events in Patients With Paroxysmal Nocturnal Hemoglobinuria (PNH): A Nested Case-Control Study in the International PNH Registry |
| **Acknowledgements**   - - Alexion, AstraZeneca Rare Disease sponsored this study. Alexion, AstraZeneca Rare Disease would like to thank all patients, their families, physicians, and patient organizations for their assistance with the International PNH Registry. The authors thank the patients and their physicians and families for participation in and support for this study.   - This summary was prepared by Jennifer Fetting, PhD, of The Curry Rockefeller Group, Tarrytown, NY, USA. Funding for preparation of this summary and editorial review was provided by Alexion, AstraZeneca Rare Disease. Patient representative(s) for PNH also provided editorial review. The original authors of the full article reviewed and approved the summary. |
